# A Comprehensive Review of Recent Trends in Posterior Malleolus Fracture Management

**DOI:** 10.7759/cureus.72081

**Published:** 2024-10-21

**Authors:** Sunandan Datta, Bratati Bandyopadhyay, Muhammad Tahir, Gourab Bose, Siddharth Khadilkar

**Affiliations:** 1 Trauma and Orthopaedics, East Kent Hospitals University NHS Foundation Trust, Margate, GBR; 2 Trauma and Orthopaedics, Aneurin Bevan University Health Board, Newport, GBR; 3 Trauma and Orthopaedics, North West Anglia NHS Foundation Trust, Peterborough, GBR; 4 Trauma and Orthopaedics, University Hospitals Birmingham NHS Foundation Trust, Birmingham, GBR

**Keywords:** ankle fractures, clinical outcomes, fixation methods, posterior malleolus fractures, surgical management

## Abstract

Posterior malleolus fractures (PMFs) are challenging injuries around the ankle that can lead to poor clinical outcomes as they can compromise ankle stability. Although there has been an evolution in the principles of management of PMFs, their optimal treatment remains controversial. This review article aims to provide an in-depth account of the management of PMF, thereby providing a better understanding of these complex cases hence resulting in improving patient outcomes.

## Introduction and background

Posterior malleolus fractures (PMFs) are relatively common, with an incidence ranging from 7% to 44% of all ankle fractures [1]. These fractures predominantly occur in the seventh decade of life, particularly among elderly women [2].

Multiple studies have revealed that rotational ankle fractures with a concomitant PMF are usually associated with poorer clinical outcomes compared to those without such a component. This is due to the attachment of vital structures that provide ankle stability, which in turn are major contributors to the syndesmosis [3].

PMFs can lead to ankle joint instability, potentially resulting in ankle joint subluxation. Orthopaedic surgeons may overlook PMFs or underestimate the size of the fracture fragment, as it is often less noticeable on lateral radiographs [4]. This necessitates additional investigations and may require supplementary surgical approaches, including both medial and lateral approaches in cases of trimalleolar fractures.

The treatment of these injuries has always been controversial. Although guidelines exist regarding the morphology and classification of these fractures, there is still no clear consensus on the most appropriate way to manage them [5].

This review article examines current practices in the management of PMFs.

## Review

Anatomy

The ankle joint is a hinged synovial joint formed by the articulation of the talus, tibia, and fibula. The ankle is stabilized by strong collateral ligaments on both the medial and lateral sides.

In 1932, Henderson described the posterior malleolus as "the anatomic prominence formed by the posterior inferior margin of the articulating surface of the tibia" [6]. He was the first to describe the term trimalleolar fracture.

The distal tibiofibular syndesmosis is made up of some vital soft tissue structures that are essential for ankle stability. The anterior inferior tibiofibular ligament (AITFL), the posterior inferior tibiofibular ligament (PITFL), transverse ligament, and interosseous ligament (IOL) form the syndesmosis [7]. A cadaveric study by Ogilvie-Harris et al. revealed that 42% of syndesmotic stability is provided by the PITFL which is by far the strongest component of all the four ligaments. The remaining 35% of integrity is provided by the AITFL and 22% by the IOL [8]. The PITFL stretches from the posterior malleolus to the posterior eminence of the fibula. This is why a PMF disrupts the structural integrity of the posterior syndesmotic ligaments, which causes syndesmotic disruption and subsequent ankle instability.

Biomechanics

The complex anatomy of the tibiotalar joint and its interaction with static and dynamic stabilizers mutually impact load characteristics. Numerous studies have examined the effects of PMFs on the biomechanics of the ankle joint, assessing stability and contact stresses. Scheidt et al. noted that PMFs covering 25% of the articular surface could potentially result in excessive internal rotation and posterior instability in a loaded ankle joint. Although they advocated that PMF fixation improved ankle stability, this was not found to be statistically significant [9].

Conversely, another perspective emphasizes the importance of the fibula and AITFL as the primary restraints to the posterior translation of the talus. Raasch et al. demonstrated that posterior subluxation of the talus in fractures involving more than 30% of the posterior malleolus occurred only after the fibula and AITFL were compromised [10]. Additionally, Harper and Hardin found that the primary factors leading to the posterior translation of the talus were the lateral ligaments and the fractured fibula, not the PMF in isolation, until 50% of the articular surface was involved [11].

Radiography and classification

A PMF is best appreciated on a lateral radiograph of the ankle; however, these fractures can be deceptive in conventional radiographs. It is standard practice to perform computed tomography (CT) scans of the ankle with 3D reconstruction in suspected cases. CT scanning provides crucial insights into fragment size, comminution, articular impaction, and syndesmotic disruption, making it an indispensable tool for accurate evaluation and treatment planning [12].

**Figure 1:**
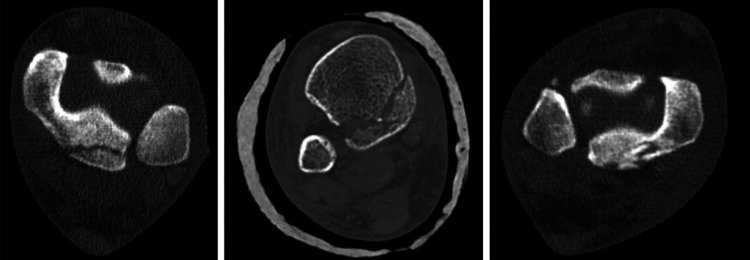
Haraguchi et al.'s classification Image Credit: [13]; published with permission

The first CT-based classification as shown in Figure 1 and Table 1 was introduced by Haraguchi et al., categorizing PMFs into three distinct categories [13].

**Table 1:** Haraguchi et al.'s classification Table Credit: [13]

Type	Description
Type 1	A posterolateral-oblique fracture with a wedge-shaped fragment, involving the posterolateral corner of the tibial articular surface
Type 2	A transverse fracture line extending from the fibular notch to the medial malleolus
Type 3	A small, shell-like fragment localized to the posterior margin of the tibial plafond

**Figure 2:**
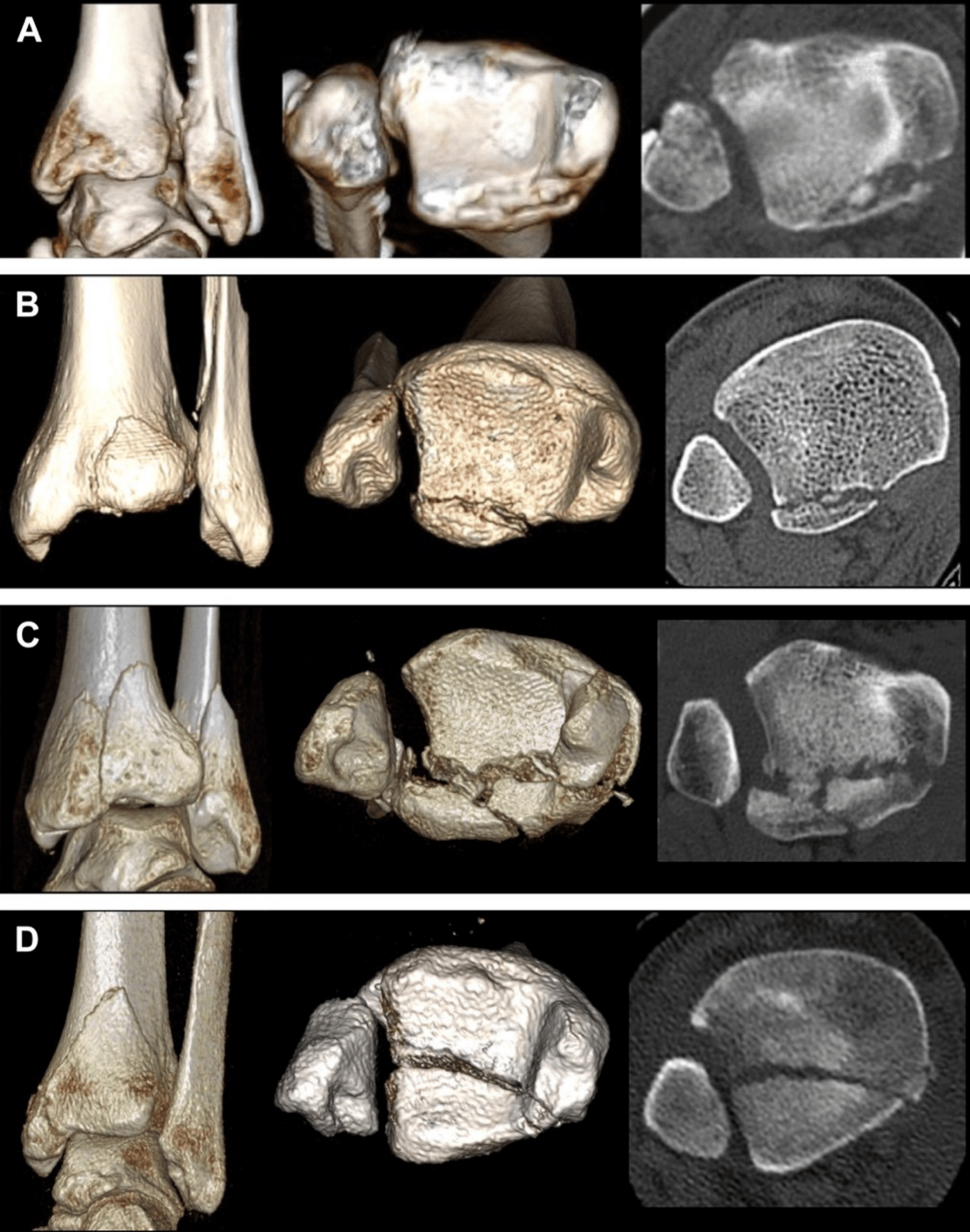
Bartoníček et al.'s classification Image Credit: [14]; published with permission

Bartoníček et al. suggested an alternative classification system that identifies five distinct fracture patterns based on CT imaging as shown in Table 2 and Figure 2 [14].

**Table 2:** Bartoníček et al.'s classification Table Credit: [14]

Type	Description
Type 1	A fracture involving an extra-incisural fragment, with an intact fibular notch
Type 2	A posterolateral fragment that extends into the fibular notch
Type 3	A posteromedial two-part fracture that includes the medial malleolus
Type 4	A large, triangular fragment located on the posterolateral aspect of the tibial plafond
Type 5	Irregular, osteoporotic fracture fragments that are difficult to classify

In Table 3, Mason et al. proposed a classification system for PMFs, dividing them into four distinct types based on the results of CT scans [15].

**Table 3:** Mason et al.'s classification Table Credit: [15]

Type	Description
Type I	A small extra-articular avulsion of the posterior malleolus
Type IIA	A posterolateral triangular fragment extending up to the fibular notch
Type IIB	A posterolateral and posteromedial fragment that subtends a 45° angle with the main fragment
Type III	A coronal plane fracture that involves the entire posterior articular surface

Principles of treatment

Historically, the crucial factor in deciding surgical intervention was the size of the posteromedial fragment, the extent of its articular surface, and its displacement [16]. As hypothesized by Scheidt et al., fragment displacement of more than 2 mm and involvement of one-fourth to one-third of the articular surface on lateral radiographs were indications for surgery [17,18]. However, the current literature lacks sufficient high-quality evidence to definitively support this threshold. Isolated, undisplaced PMFs should be treated nonoperatively. Several studies have shown excellent outcomes with conservative treatment due to the intact fibula and lateral ligaments [19].

Currently, surgical decisions are based on the morphology of the PMF rather than solely the size of the fragment. Preoperative radiographs and CT scans should be thoroughly analyzed to gain vital information about specific fracture geometry, including the amount of articular incongruity, presence of loose bodies, or articular impaction, which should all be considered during surgical planning [20].

Strategies for fixation

The optimal position for fixation of these injuries is either a lazy lateral or a staged prone and supine position. In a trimalleolar fracture, the sequence of fixation typically begins with PMF fixation, as the fibular plate can obstruct visualization and assessment of posterior malleolus reduction during intraoperative fluoroscopy. Direct reduction (from posterior to anterior) is preferred over indirect reduction, as it allows for the precise reduction of impacted fragments or the removal of comminuted loose bodies [4].

The posteromedial approach is suitable for fractures involving the posteromedial fragment (or a two-part PMF with medial extension, Haraguchi et al. type 2) and allows for the simultaneous treatment of the medial malleolus fracture. This approach utilizes the interval between the tibialis posterior tendon and flexor digitorum longus tendon [4]. In addition, the posteromedial approach is flexible since the surgeon can adjust according to the placement of the bone fragment and one can pull all tendons laterally and can use the interval between the tibialis posterior and flexor digitorum longus and also use the interval between the flexor digitorum longus, flexor hallucis longus, and neurovascular bundle.

Recently, the posterolateral approach has gained popularity as it provides excellent visualization of the posterolateral malleolus fragment and allows for the concurrent fixation of the fibular fracture. The posterolateral approach uses the interval between the peronei and the flexor hallucis longus. Protection of the sural nerve is crucial during this approach, which significantly reduces the risk of encountering the posterior neurovascular bundle, making it a safer option [4]. Both approaches typically utilize prone positioning, with the feet extending just beyond the operating table.

A study by Miller et al. suggests that prone positioning and direct fixation of the PMF can effectively reduce syndesmotic instability in most trimalleolar fractures [21].

These fractures can be fixed with posterior-to-anterior screws parallel to the joint line or using a buttress plate, which employs an anti-glide technique that aids in fracture reduction. O'Connor et al. observed that patients undergoing posterior buttress plating had superior clinical outcomes compared to those fixed with anterior-to-posterior screws. Anti-glide plating is effective in stabilizing PMFs as these are partial articular fractures, negating the shearing forces that often lead to displacement [22].

Postoperative management

In general, patients are kept non-weight-bearing in a posterior plaster splint for approximately two weeks post-surgery. After a wound check at two weeks, the postoperative plan may vary depending on the morphology of the fracture, stability of the construct/quality of fixation, and patient factors such as comorbidities and pre-injury mobility status. Wounds around the ankle have a higher risk of infection, dehiscence, and delayed healing, particularly in patients with diabetes mellitus, pre-existing peripheral vascular disease, and a history of chronic smoking. For most patients, allowing weight-bearing as tolerated in a pneumatic or walking boot, along with early range of motion exercises, is adequate.

Early weight-bearing has been demonstrated to be safe in those with syndesmotic injury, as multiple studies have indicated no posterior instability following trimalleolar ankle fracture fixation [23].

Clinical outcomes

There has been a steady evolution in the management of trimalleolar ankle fractures over the past few decades, with a consensus emerging around the importance of accurate anatomic reduction and stable fixation of the ankle joint. Although there remains some ambiguity regarding the routine fixation of all PMFs, increasing evidence supports the routine fixation of most PMFs [15,18].

Historically, emphasis was placed on the percentage of tibial plafond involved; however, newer studies focus more on fracture geometry and postoperative articular congruity as key factors in achieving better clinical outcomes [24]. 

A randomized controlled trial by Karaismailoglu et al. revealed that patients with an articular step-off greater than 1 mm experienced poor functional outcomes [25]. Shi et al. compared the direct posterolateral approach to the indirect approach (anterior-to-posterior screws) for the fixation of PMFs involving more than 25% of the tibiotalar articular surface. They found that the quality of reduction was better in the direct reduction and fixation group, with higher American Orthopaedic Foot and Ankle Society (AOFAS) scores and no significant difference in range of motion or Visual Analog Scale (VAS) scores [26].

Langenhuijsen et al. concluded that for all PMFs with articular involvement of more than 10%, the key to superior clinical outcomes was the restoration of joint congruity, regardless of whether fixation was performed. Only those fragments that were displaced after lateral and medial malleolus reduction were considered for fixation [27].

A study conducted by Xu et al. investigated the correlation between the size of the posterior malleolar fragment and functional outcomes. PMFs were divided into three groups based on articular involvement on lateral radiographs: less than 10%, 10-25%, and greater than 25%. While the AOFAS and VAS scores were similar across all three groups, patient satisfaction was lowest in the group with PMFs less than 10% and highest in the 10-25% group. The highest prevalence of arthritis was found in the group with fractures exceeding 25% [28].

Verhage et al. demonstrated that a persistent articular step-off greater than 1 mm was a significant risk factor for the development of post-traumatic arthritis. It was also noted that AOFAS scores were significantly poorer for patients with evidence of osteoarthritis and high body mass index (BMI) [29].

Plate fixation has been shown to be a superior method compared to screw fixation. A meta-analysis by Tu et al. concluded that, compared to screw fixation, plate fixation resulted in significantly longer surgery times and more intraoperative blood loss but also led to shorter hospital stays, higher AOFAS scores, and better Baird-Jackson scores. Furthermore, plate fixation was associated with faster bone healing, earlier achievement of full weight-bearing, quicker off-bed mobilization, reduced postoperative pain, lower complication rates, decreased loosening rates, fewer instances of malunion, and a reduction in postoperative osteoarthritis [30].

In addition to stiffness, common complications include post-traumatic arthritis, wound complications, implant failure, and screw loosening. Medial or lateral forefoot paresthesia remains a relatively uncommon adverse effect. Foot and ankle surgeons who frequently perform these procedures are generally adept at employing these techniques, achieving excellent outcomes with minimal complications.

## Conclusions

PMFs present a significant challenge for fixation due to their complex anatomy and difficult visualization. The routine use of CT scans has facilitated the accurate identification of fracture morphology, making it essential for surgical planning to consider the extent of articular involvement and the presence of impaction and loose bodies. There is a clear consensus that the fixation of PMFs considerably improves the rotational stability of the ankle.

Our approach to these fractures has evolved significantly over the past few decades as we have gained a better understanding of ankle joint biomechanics, with articular congruity identified as the most critical factor for successful outcomes, regardless of the size of the PMF. Despite the overall improvement in clinical outcomes due to enhanced understanding and techniques, ankle injuries involving posterior malleolus fractures remain challenging to treat. Therefore, meticulous surgical planning is essential for optimal results.
